# Exploration of spatial clustering in maternal health continuum of care across districts of India: A geospatial analysis of demographic and health survey data

**DOI:** 10.1371/journal.pone.0279117

**Published:** 2022-12-15

**Authors:** Mohd Usman, Umenthala Srikanth Reddy, Laeek Ahemad Siddiqui, Adrita Banerjee

**Affiliations:** 1 Indian Council of Medical Research (ICMR)-National Institute of Cancer Prevention and Research (NICPR), Noida, India; 2 Department of Public Health and Mortality Studies, International Institute for Population Sciences, Mumbai, India; 3 Department of Biostatistics and Epidemiology, International Institute for Population Sciences, Mumbai, India; Indian Institute of Dalit Studies (IIDS), INDIA

## Abstract

**Introduction:**

The continuum of care (CoC) throughout pregnancy, delivery and post-delivery has recently been highlighted as an integrated intervention programme for maternal, new-born, and child health. Existing literature suggests the importance of continuum of care (CoC) for improved maternal and child health outcomes. However due to unavailability of data at the lowest administrative levels, literature on spatial pattern of uptake of full CoC is lacking. The present study attempts to focus on the spatial analysis of CoC in maternal health care in India.

**Data and methods:**

The study is based on the fourth round of National Family Health Survey data conducted in 2015–16 in India. The outcome variable used is maternal health continuum of care which includes- at least 4 ANC visits, delivery through skilled birth attendant and postnatal check-up within 48 hours of delivery. Univariate and bivariate Local Indicator of Spatial Association (LISA) maps have been generated to show the spatial pattern of CoC across 640 districts in India. We also employed spatial regression techniques to explore the determinants of CoC.

**Findings:**

Percentage of women who followed full CoC was observed to be least for East Kameng (0.0%) district of Arunachal Pradesh and highest in North Goa district (90.4%). Majority of districts where uptake of full CoC was more than 80 percent were found concentrated in southern region on India. Equivalently, findings indicated a strong spatial clustering of full CoC with high-high clusters mostly concentrated in southern districts. Low-low district clusters are concentrated in the states of Uttar Pradesh, Bihar and Madhya Pradesh. For complete CoC the global Moran’s I is 0.73 indicating the spatial dependence. The spatial regression analysis suggested that modern contraceptive use, meeting with health worker, urbanization and secondary or above education for women have positive impact on the utilisation of CoC.

**Conclusion:**

The spatial pattern indicates district level clustering in uptake of CoC among women. The study suggests policymakers and stakeholders to implement comprehensive interventions at sub-regional levels for ensuring the completion of CoC for women which acts as a preventive measure for adverse outcomes such as-maternal and child mortality.

## Introduction

Maternal health and wellbeing facilitate in measuring the progress of a country in terms of increasing equity and reducing poverty. The survival and wellbeing of mothers is not only important for the children and family, but are also central in explaining broader economic, social and developmental challenges. Mostly maternal and new-born deaths are due to inadequate maternal health care from pregnancy to childbirth. Although, the UN SDG3 aims in reducing maternal mortality to 70 per 100000 live birth by 2030, in 2017, globally, about 300000 women died due to maternal health complications and 90% of these deaths were in low and middle income countries (LMIC) [1]. This shows the lack of public health system in these LMICs. What is needed is a high-quality health system that optimise the health care needs of the people. A system that is valued and trusted by people and can easily respond to changing population needs.

Till recently, the success of maternal health care was measured by the coverage of individual indicators namely antenatal care (ANC), skilled birth attendance (SBA) and post-natal care (PNC) separately [2,3]. These three services are indeed essential for reducing pregnancy complications and maternal mortality [4]. Existing literature suggests that continuous uptake of ANC, delivery with SBA, and PNC is necessary to improve MNCH outcomes in low- and middle-income countries [5,6]. But, access of all three of these in a sequential way may be more effective [7]. The importance of these three interventions calls for an integrated approach in maternal and child health care needs. Given the need for an optimal public health system on which the population can trust, the continuum of care (CoC) has recently been highlighted as an effective intervention programme for maternal, new-born, and child health.

Initially the term ‘Continuum of care (CoC)’ was introduced in 1970s and was applied for research and practice on elderly people [8]. But in subsequent years the use of the term expanded and has recently been used in maternal and child health (MNCH) [9,10]. CoC is a vital program that helps to reduce the burden of half a million maternal deaths, 4 million neonatal deaths, and death of 6 million children below the age of 5 years [11]. Availing the three services also lowers adverse neonatal and perinatal outcomes [5,12–14]. Continuum of care in maternal health has two key dimensions, time and place, time advocates the delivery of essential maternal health services at each stages from pregnancy to postpartum periods, whereas dimension of place ensure the levels of care at home, community, and health facilities [14,15].

Recently, public health research has started focussing on understanding the health of the population in different geographical regions or space using geospatial analysis [16–19]. Few attempts have been made to study the spatial correlation of individual maternal health indicators. For example, Paul [20] attempted to analyse the spatial heterogeneity in PNC in India. The author found a lower use of PNC in the north, central, east, and northeast regions, while PNC utilization was higher in southern, followed by western region of the country. Similarly, a study on the determinants of antenatal care utilization in India found spatial dissimilarities in ANC utilization by women and a moderate spatial autocorrelation (Moran’s I = 0.6210) confirmed the spatial dependence of ANC [21]. A few other studies have also observed significant spatial heterogeneity in ANC [22,23], institutional delivery [24], and PNC [25–27]. Studies have also observed that home delivery after ANC was spatially clustered [28,29]. To our knowledge, none of the published studies attempted to study the spatial dependence of maternal health service utilization with an integrated approach considering CoC. The presence of significant spatial correlation in maternal health services observed in existing literature highlights the need of conducting studies that attempt to fill this gap in literature.

India accounts for one of the highest numbers of maternal deaths and hence implementation and evaluation of programs designed to improve maternal and child health is much needed.

But only few studies have analysed the utilisation of CoC in terms of impact on child health and many studies have analysed the components of CoC like ANC, institutional births and Post-natal care as different components of maternal care utilisation in Indian context. Thus existing literature in Indian context have dealt with the three approaches separately and have always overlooked the integrated approach [30–32]. Studies on CoC in India are very few and only have dealt on the impact of continuum of care on child health [14,33,34]. Studying the spatial pattern of uptake of CoC is very important from policy perspective as it would help in locating the areas where uptake of all components of CoC is low. However, literature on this concept is scarce and inconclusive as data at the lowest administrative levels which would show a strong spatial association was not available earlier. Hence, the present study has tried to focus on the spatial analysis of CoC in maternal health care at district level in India. District is the lowest level of administrative region in India, identifying the hotspots of CoC at a lowest level of administrative may help in allocation of programme resources properly.

## Data and methods

### Data

The data for this study has been utilized from fourth round of National Family Health Survey (NFHS-4) for the period 2015–16. NFHS-4 is a nationally representative cross-sectional household survey, primarily conducted to provide reliable estimates of fertility, mortality, and various maternal and child care indicators at national, state and district levels in India. The analytical sample consists of 190,898 women of age 15–49 who had their most recent birth in last 5 years. In the fourth round of NFHS, the information regarding antenatal and postnatal care of women has been obtained only for last birth in 5 years preceding the survey [34]. The unit of analysis is the districts of India whereby data is available for 640 districts. The TFR for the country is 2.2. The TFR ranges from 0.9 births per women in Chennai district of Tamil Nadu to 5.66 births per women in Mewat district in Haryana (S1 Table).

#### Ethics and data availability statement

This study is based on datasets that are available in public domain. The data can be accessed from the Demographic and Health Survey (DHS) website after obtaining permission. The link to the site is https://dhsprogram.com/data/dataset/India_Standard-DHS_2015.cfm?flag=0. DHS follows all necessary ethical protocol while collecting the data. The dataset does not have any identifiable information of the survey participants. Hence, no ethical approval is required for the current study.

#### Outcome variable

The outcome variable for the study is maternal health Continuum of Care (CoC). A mother is said to be following the maternal health continuum if-

1. She had at least 4 ANC visits for her last birth
2. Her delivery was assisted by a skilled birth attendant (i.e., doctors, auxiliary nurse, midwives, nurses, midwives, and lady health visitors)
3. She had a postnatal check-up within 48 hours of delivery

We followed the standard definition of maternal health CoC from earlier published literature [6,7].

#### Independent variables

In India, the maternal health utilization has been observed to be significantly associated with religion, caste and educational and financial status of women [34–36]. Women who are followers of Islam and those belonging to marginalized caste groups (such as Scheduled castes/tribes) are less likely to utilize maternal health services as compared with Hindu women and those from ‘General’ caste groups [37–39]. The Scheduled Castes (SCs) and Scheduled Tribes (STs) are officially designated groups of people and among the most disadvantaged socio-economic groups in India. Findings of previous literature indicate towards a clear relationship between low maternal health service utilization and early marriage of women [40–42]. Studies have also found modern contraceptive use to be significantly associated with maternal health services utilization [43]. Thus based on earlier literature the specific independent variables included in the analysis for each of the 640 districts were- percentage of women married before the age of 18, percentage of Hindu women in the reproductive ages (15–49 years), percentage of women belonging to Scheduled Caste (SC) or Scheduled Tribes (ST), percentage of women who have completed secondary or more education, percentage of women using modern contraception, percentage of women who belong to poorest households and percentage of women living in urban areas. Among other variables percentage of women who met with any health worker in last 3 months was also included in the analysis as a proxy of exposure of maternal health services through health workers.

### Statistical methods

Geospatial analysis accounts for the effect of spatial diffusion in particular health or socioeconomic outcomes. It also aims to test spatial correlations between variables, i.e. whether an inter-relation can be found between the spatial distribution of outcome variable and a range of other variables [44,45]. To show the district wise spatial pattern of CoC indicators, descriptive maps were generated. For each of the CoC indicators (i.e., ANC visits, skilled birth attendance and post-natal care) we prepared choropleth maps to show the spatial pattern of each of these indicators across districts of India (S1 Fig). Similar map was generated for full CoC (Fig 2). We used the ARC-GIS 10.1 version to generate all the maps for this analysis.

The first step in spatial analysis is to identify the spatial dependence in the variable of interest. For this purpose, both global and local indicators were used. To measure the overall spatial clustering in CoC, we computed Moran’s I index. Moran’s I is the correlation coefficient that measures the overall spatial autocorrelation in the dataset. The value of Moran’s I varies from -1 to +1. A positive Moran’s I value indicates observations with similar values surrounding each other- also known as spatial clusters, and a negative Moran’s I value denotes observations with high values surrounded by low values or vice-versa-called as spatial outliers [46,47]. A zero value shows a spatial randomness. In the analysis, we employed first-order contiguity matrix with rook’s criterion as our weight matrix.

Univariate Local Indicators of Spatial Autocorrelation (LISA) maps depict the autocorrelation of a particular geographical unit with the neighbourhood, while bivariate LISA measures the autocorrelation in the neighbourhood between a variable and weighted average of another variable [47].

Each LISA map depicts five possible scenarios:

- High-High scenario: that is location with high values are surrounded by the similar higher values
- Low-Low scenario: that is location with low values are surrounded by the similar lower values
- High-Low scenario that is location with high values are surrounded by the low value neighbours
- Low-High scenario that is location with low values are surrounded by high value neighbours
- Location with no significant correlation

### Spatial regression analysis

We also employed spatial regression techniques to explore the determinants of CoC. At first, the ordinary least square (OLS) model was applied to examine the relationship between CoC and the independent variables. There are certain underlying assumptions of OLS regression technique which need to be met for its estimates to be valid. However, these assumptions may not be always satisfied in practice. When a value observed in one location depends on the values observed at neighbouring locations, there is a spatial dependence. Spatial data may show spatial dependence in the variables and error terms. With spatial dependence in the variables and error term of the regression equation, the OLS assumption of uncorrelated errors is violated. As a result, the estimates are inefficient [48]. Again with spatial lag in OLS regression, the assumption of uncorrelated error terms along with the assumption of independence of observations is violated, resulting in biased and inefficient estimates [16,48]. To overcome these limitations, we applied spatial error and spatial lag models in our data to identify the determinants of CoC. A more detailed discussion on the spatial regression may be found elsewhere [16,48,49]. For detailed spatial analysis we utilized GeoDa 1.20 software.

## Results

**Fig 1:**
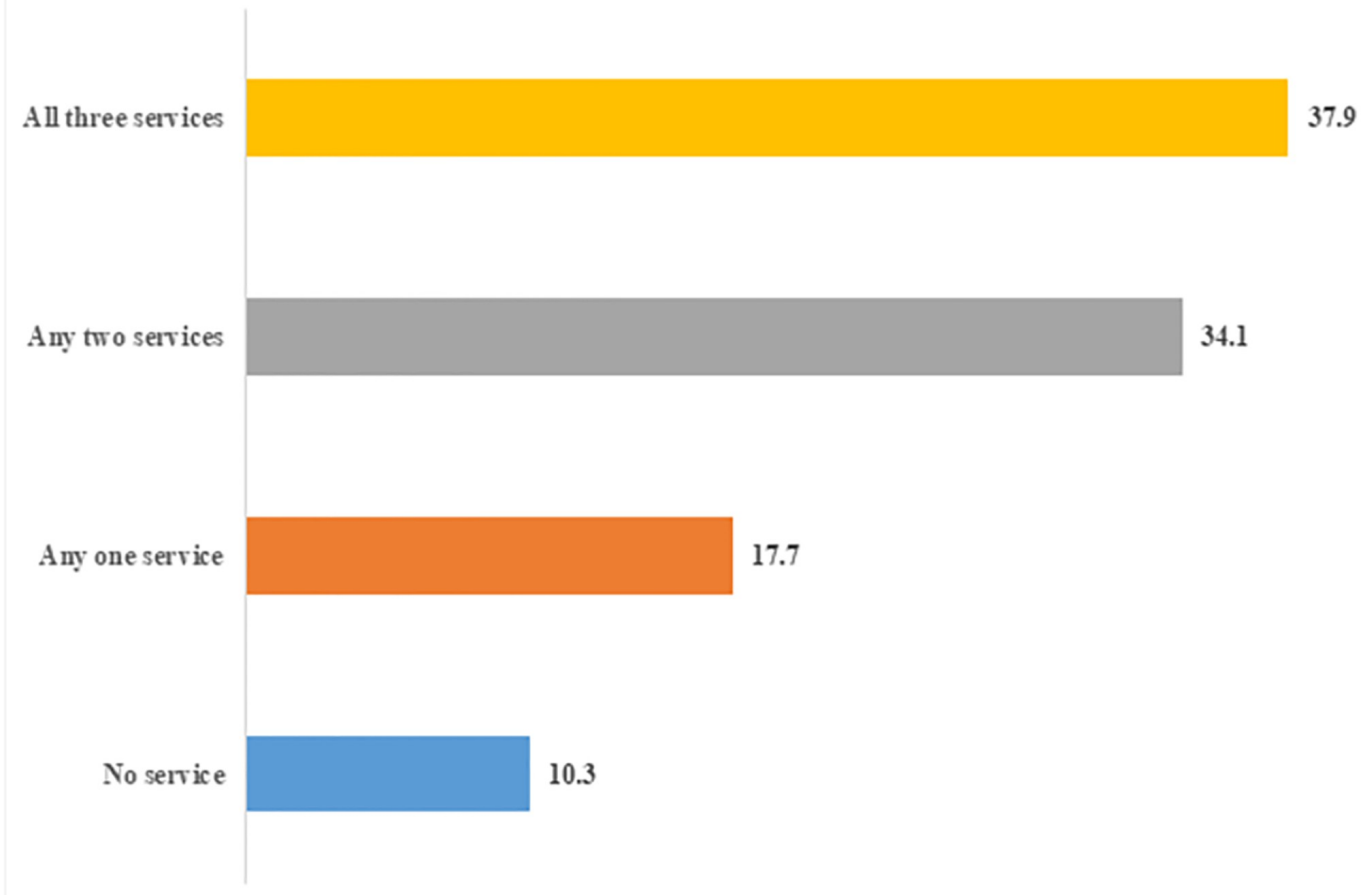
Proportion of women who utilized different number of maternal health services for their most recent birth in last 5 years.

**Fig 2:**
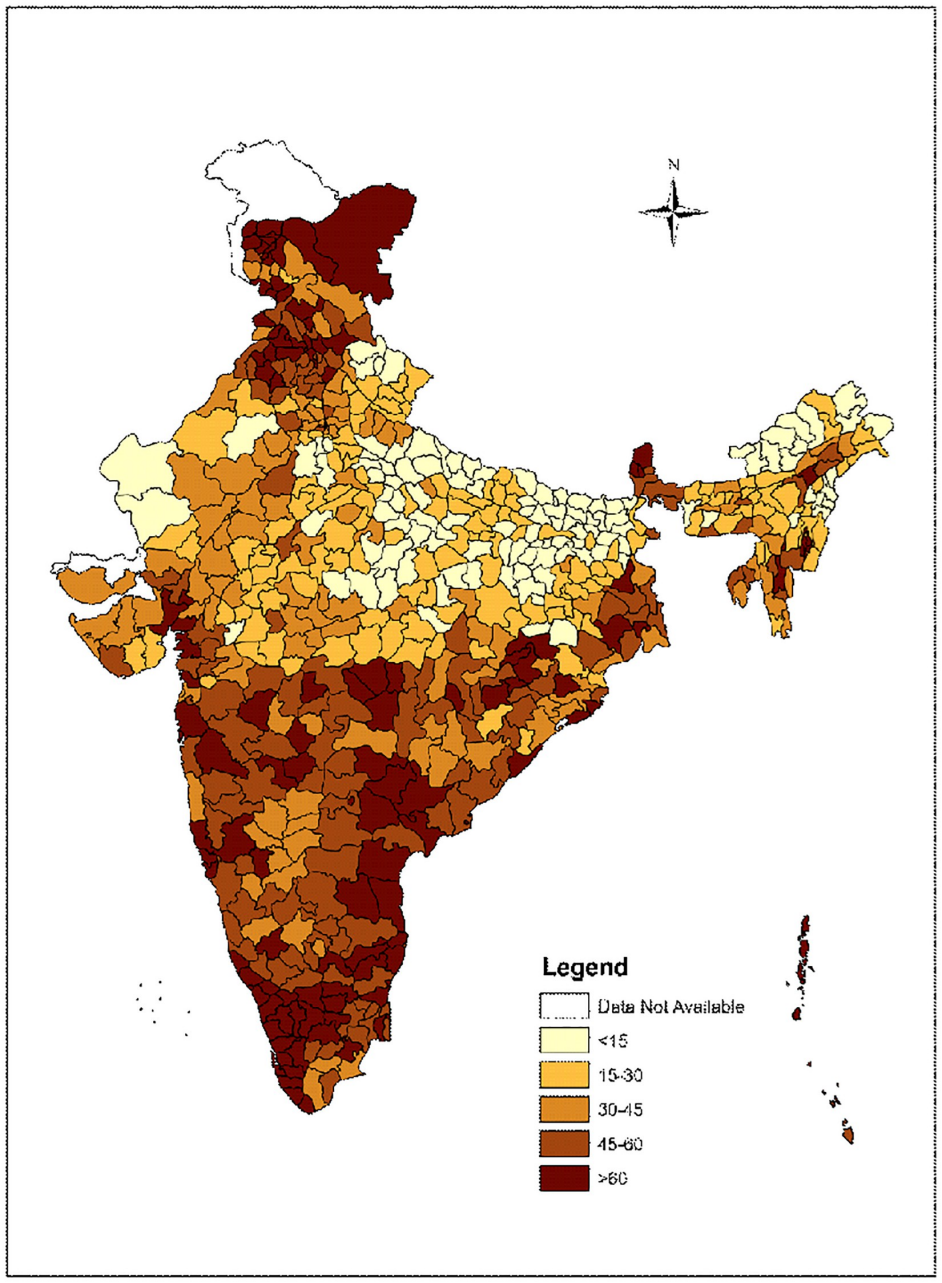
District-wise distribution of full continuum of care among mothers for their last births, India, 2015–16.

Fig 1 shows the proportion of women who utilized different number of maternal health services for their most recent birth in last 5 years. It is evident from the figure that 37.9% women in India followed the full CoC. The district wise distribution of full CoC among mothers for their last birth is shown in Fig 2. It is observed from the figure that districts with high levels (>60%) of full CoC are mostly concentrated in southern region, while districts with less than 15% of the mothers availing full CoC are concentrated in North and North-Eastern regions of India. Percentage of women who followed full CoC was observed to be least for East Kameng (0.0%) district of Arunachal Pradesh and highest in North Goa district (90.4%).

**Fig 3:**
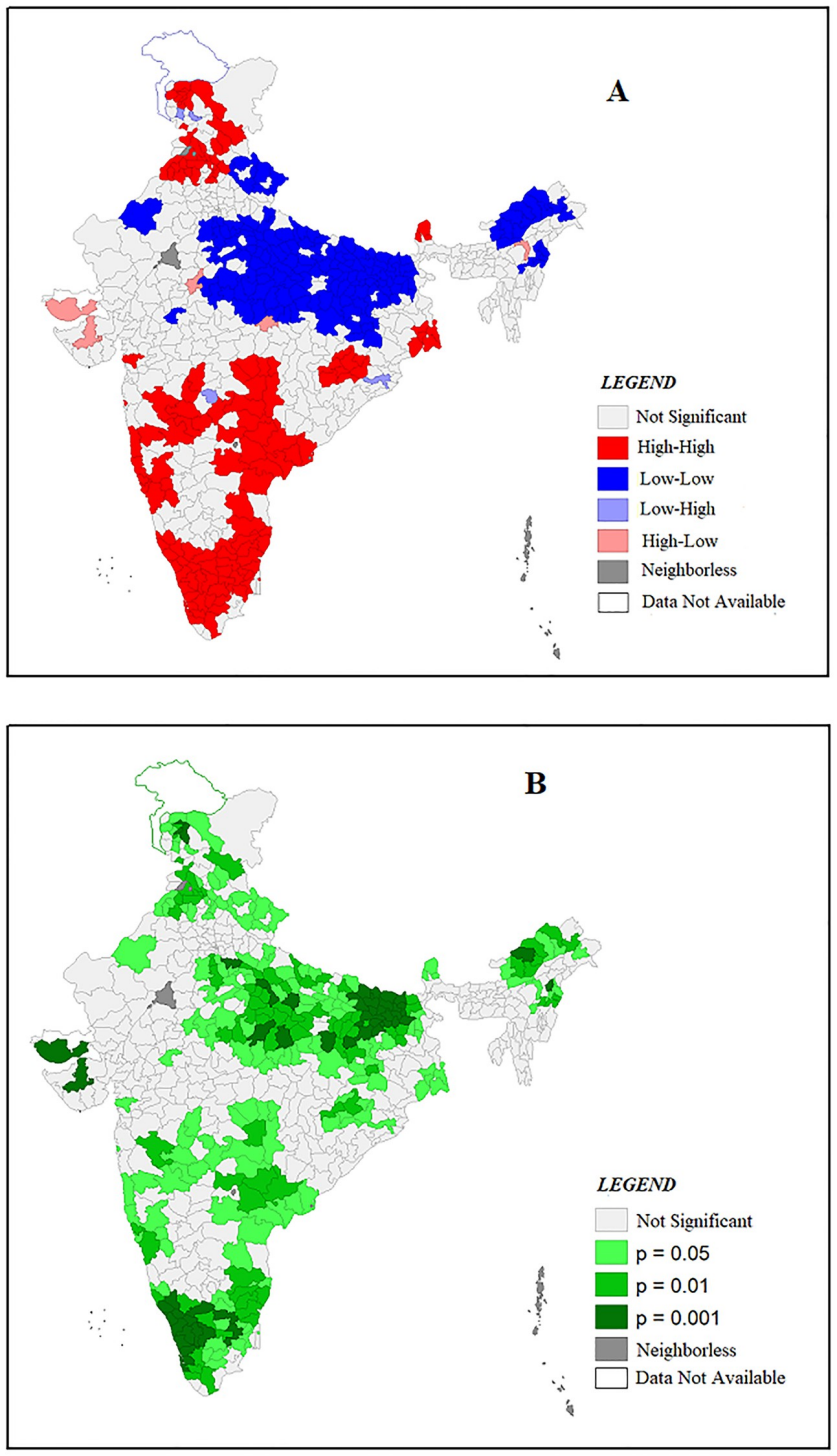
A and B: Univariate LISA Cluster and Significance Map showing Spatial Clustering and Outliers of Complete CoC.

Univariate LISA map for Continuum of Care (CoC) is presented in Fig 3. High-High clustering indicates district with high use of CoC shares boundaries with similar value of CoC. Low-Low indicates that district with low coverage of CoC share boundaries with low CoC coverage. High-low and Low-High indicate the spatial outliers, these spatial outliers indicate that district with high CoC coverage share boundaries with low CoC coverage and vice-versa. From Fig 2 it is evident that high-high clusters are mostly concentrated in southern region and also in some districts of Punjab and Himachal Pradesh. While the Low-Low district clusters are concentrated in the states of Uttar Pradesh, Bihar and Madhya Pradesh.

### Bivariate LISA

**Fig 4:**
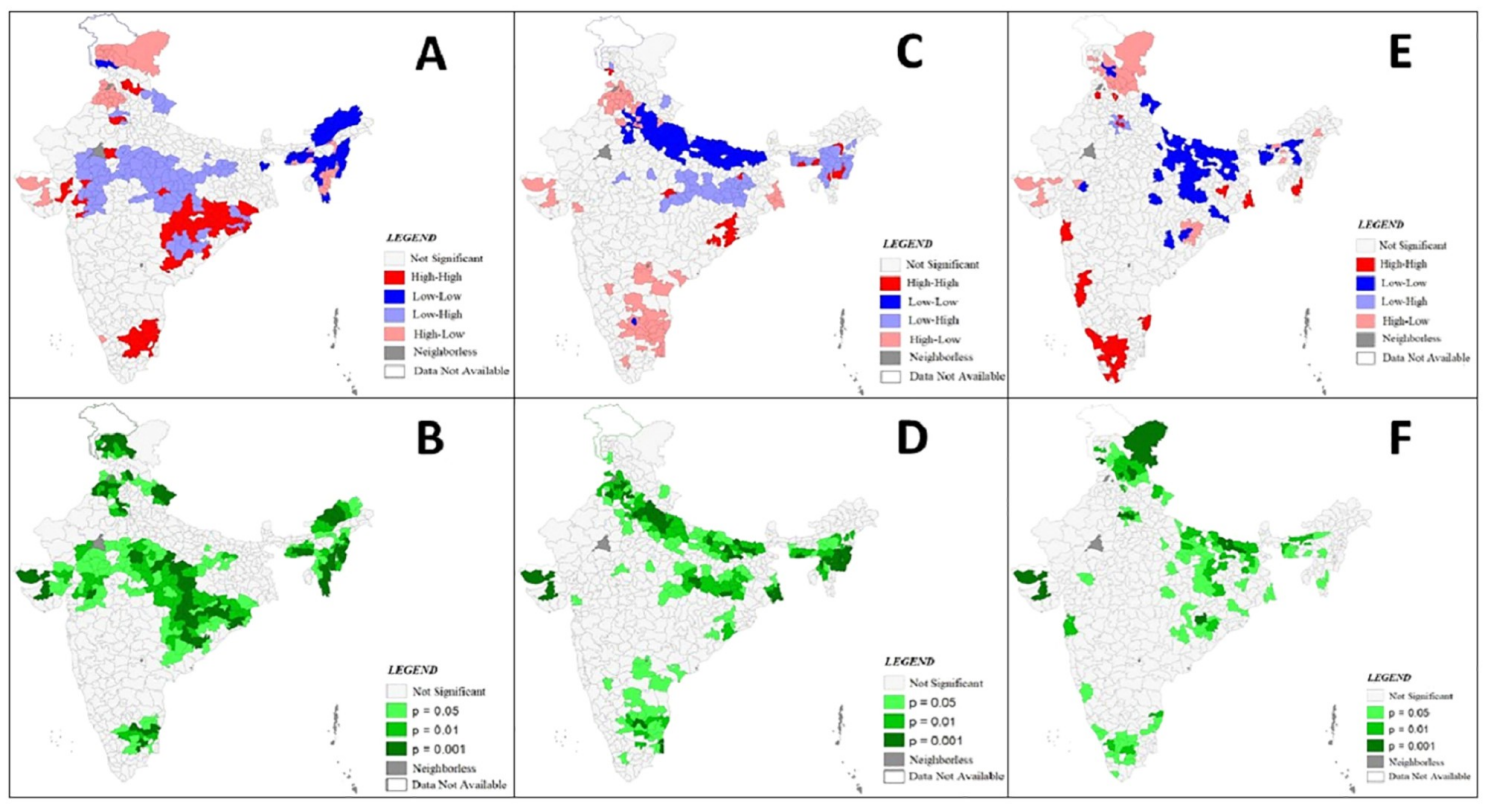
Bivariate LISA (cluster and significance) maps depicting spatial clustering and spatial outlier of continuum of care and independent variables in India.

**Fig 5:**
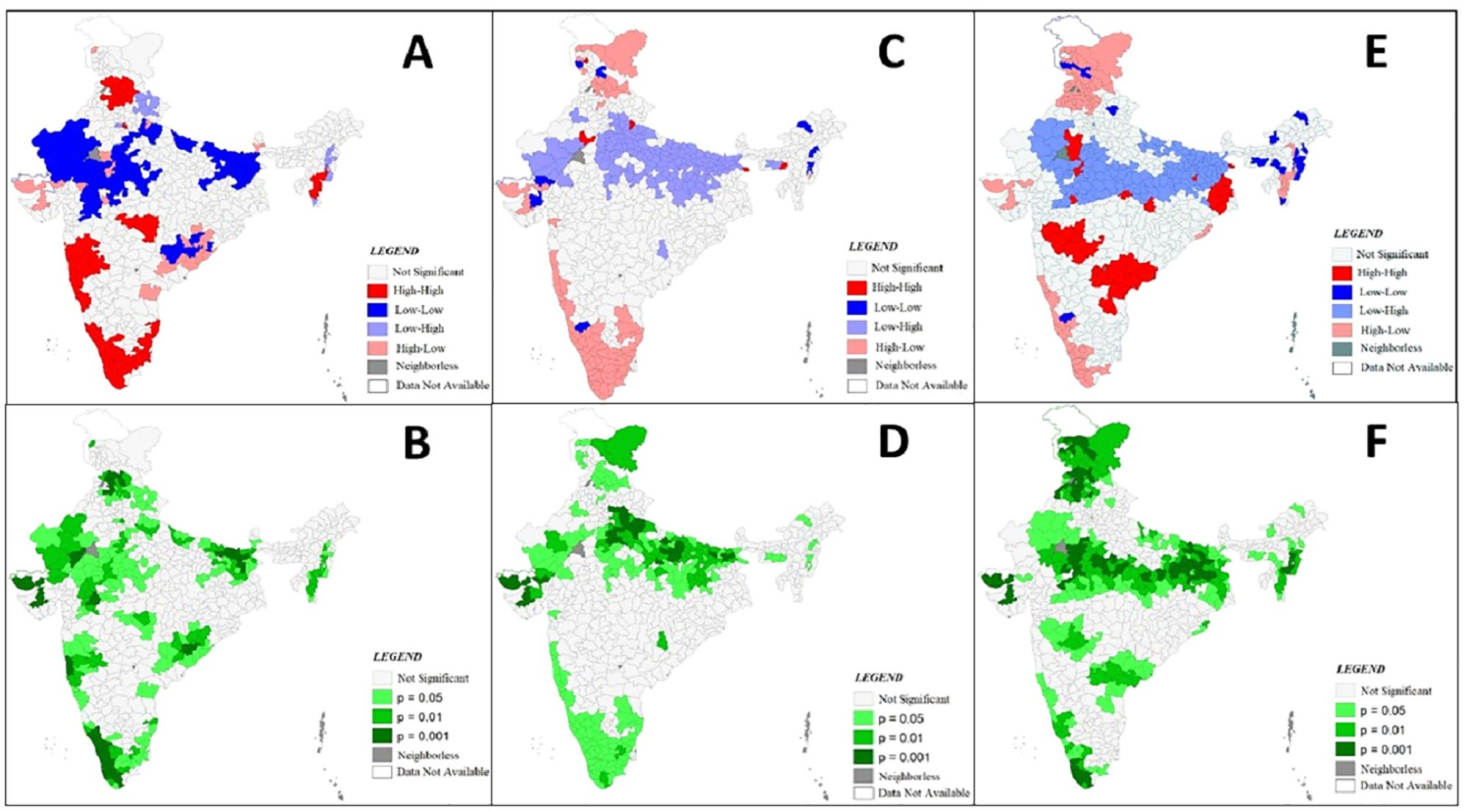
Bivariate LISA (cluster and significance) maps depicting spatial clustering and spatial outlier of continuum of care and independent variables in India.

**Fig 6:**
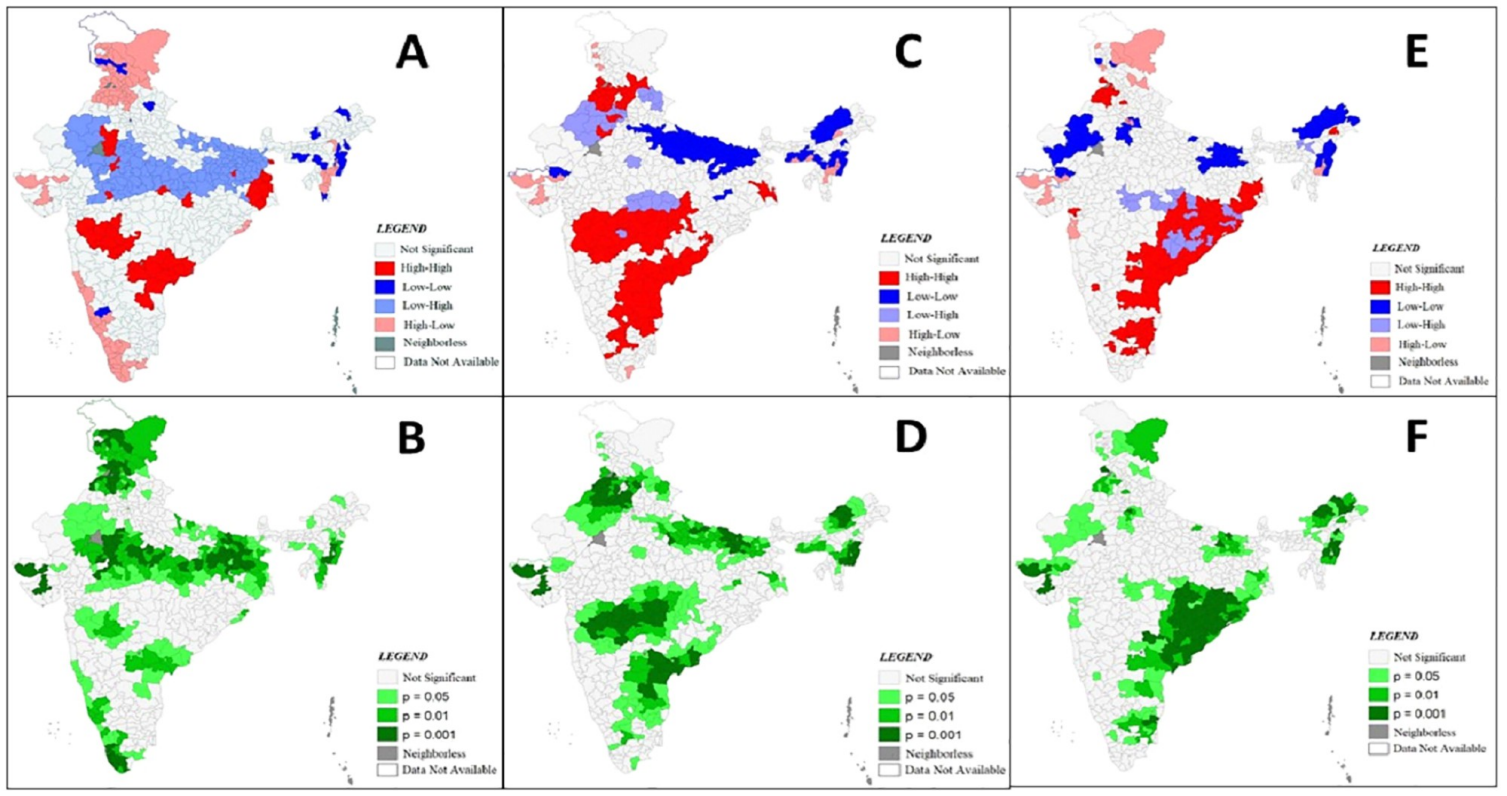
Bivariate LISA (cluster and significance) maps depicting spatial clustering and spatial outlier of continuum of care and independent variables in India.

From the univariate LISA of CoC it was evident that there is a geographical clustering of CoC in districts of India. The bivariate LISA examines the spatial relationship between CoC and independent variables. This bivariate LISA relationship examines whether the regions which were underprivileged were also disadvantaged with lower coverage of complete CoC. The figures of bivariate LISA for each independent variable and their spatial associations with the CoC has been presented in Figs 4, 5 and 6. The spatial association between percentage of Hindu women in the district, Schedule Caste and Tribe women and urban women with completed CoC are presented in Fig 4. From the Fig 4A & 4B it is evident that few districts in the states of Orissa, Jharkhand, Tamil Nadu and Chhattisgarh have a high–high association i.e. the proportion of Hindu women is high and CoC uptake is also high. However, it is seen that central part of India having higher proportion of Hindu women have lower coverage of CoC. In contrast some districts in Jammu and Kashmir have lower proportion of Hindu women, but CoC is high. Most districts of Rajasthan and some districts of Bihar showed a low-low association between poor wealth quintile and COC coverage. While, in Kerala and parts of Tamil Nadu higher complete CoC was observed in districts with lower poor quintile. In terms of education and completion of CoC as seen in Fig 5C & 5D the north-south division is clearly visible, as in regions of north, women who competed secondary and higher education have lower CoC completion, wherein in southern region districts we found a contrast. The southern regions have higher completion of CoC even though women have lower educational levels. From Fig 6C it is seen that high-high cluster i.e. districts where contraceptive use is high, uptake of full CoC is also high are concentrated in the central and south-eastern part of the country, whereas low-low clusters are concentrated in districts in states of Bihar and Uttar Pradesh. Fig 6E shows the bivariate association between meeting with health workers and uptake of CoC. Mostly we see a high-high association between the two. But in few districts of Jammu and Kashmir and parts of Gujarat there are outliers which show a high-low association, i.e. uptake of CoC is high but the women meeting health care professional is low.

### Moran’s I

After the spatial dependence examination through maps, the magnitude of this spatial dependence was calculated using Moran’s I. In this study, we estimated global Moran’s I values. For variable of interest that is completed CoC and other independent variables Moran’s I is computed and presented in Table 1. Moran’s I value ranges from +1 to -1, values closer +1 indicates similar values occurring near one another in neighbourhood and values close to -1 spatial dispersion. From the Table 1 it is clear that for all variables, the Moran’s I values are nowhere close to 0 rejecting the null hypothesis of no spatial dependence.

**Table 1:** Moran’s I value for dependent and independent variable.

Variables	Moran’s I*	
	Univariate	Bivariate
Complete Continuum of Care	0.732***	-
Hindu Population	0.777***	0.047***
SC/ST Population	0.567***	-0.125***
Urban Population	0.448***	0.266***
Poor Wealth Quintile	0.763***	-0.505***
Secondary level education	0.682***	0.436***
Age of Mother above 30 years	0.490***	-0.431***
Married below 18 years	0.795***	-0.415***
Contraceptive Use	0.709***	0.431***
Met Health Worker in last 3 months	0.636***	0.360***

*** Moran’s I Value significant at p<0.01, No of Districts = 640.

For complete CoC the global Moran’s I is 0.73, indicating a high spatial clustering in the completion of CoC among women for their most recent birth in last 5 years. For percentage of women married below 18 years, a higher spatial clustering was observed (Moran’s-I = 0.79). A lower clustering value is found in urban population (Moran’s-I = 0.448) and women of age above 30 years of age (Moran’s-I = 0.49). The results indicate that there is substantial spatial autocorrelation even in independent variables included in this analysis.

### OLS and spatial error model

Spatial regression models may be viewed as a generalisation for linear regression after accounting for spatial autocorrelation among the variables. From diagnostics (Table 2 AIC value and S2 Table) it is evident that spatial error model is an appropriate model for accounting of spatial autocorrelation. In Table 2 we presented ordinary linear regression (OLS) and spatial error model (SEM) results. OLS model explains 69% of the variation in the Continuum of Care. After accounting for spatial autocorrelation in the SEM regression, we found that SEM model explains 83% of the variation in CoC. Lambda, the spatial dependency parameter is statistically significant, which indicates that there exists spatial dependency. SEM model and OLS have similar effects on the utilisation of CoC among the district of India. In the SEM model percentage of Hindu women population, percentage of urban population, percentage of women who have above secondary education, modern contraceptive use and meeting with health worker have positive impact on the utilisation of continuum of care. With 1 percent increase in meeting with health worker and use of contraception increases the utilisation of CoC by 0.35 and 0.31 respectively. Variables such as percentage of schedule caste and schedule tribe, mothers above 30 years of age, and married below 18 years of age have negative impact on the utilisation of CoC after controlling for other variables.

**Table 2:** Results of spatial OLS model, spatial lag model and spatial error model of complete COC for India, 2015–16.

Variables	Spatial OLS Model			Spatial Lag Model			Spatial Error Model		
	Coefficient	Std. Error	Probability	Coefficient	Std. Error	Probability	Coefficient	Std. Error	Probability
Hindu Population	0.102	0.022	0.000	0.082	4.232	0.000	0.060	0.026	0.020
SC/ST Population	-0.089	0.050	0.077	-0.088	-2.017	0.044	-0.060	0.048	0.207
Urban Population	0.122	0.031	0.000	0.108	3.999	0.000	0.076	0.029	0.010
Poor Wealth Quintile	-0.159	0.039	0.000	-0.110	-3.246	0.001	-0.172	0.043	0.000
Secondary level education	0.330	0.049	0.000	0.288	6.767	0.000	0.350	0.053	0.000
Age of Mother above 30 years	-0.560	0.104	0.000	-0.245	-2.681	0.007	-0.054	0.098	0.580
Married below 18 years	-0.069	0.064	0.283	-0.001	-0.013	0.990	-0.070	0.068	0.301
Contraceptive Use	0.258	0.042	0.000	0.168	4.470	0.000	0.312	0.041	0.000
Met Health Worker in last 3 months	0.475	0.034	0.000	0.347	11.216	0.000	0.336	0.034	0.000
Constant	15.380	5.797	0.008	-5.256	5.137	0.306	-3.468	4.538	0.044
Number of Observations	640			640			640		
Log Likelihood	-2524.060			-2449.990			-2381.76		
AIC	5068.120			4921.97			4783.54		
R square	0.938			0.766			0.830		
Lag Coefficient (RHO/Lambda)				0.382			0.689	0.030	0.000

## Discussion

Continuum of care is an integrated approach proposed as a key framework for the delivery of maternal, neonatal and child health services. The two dimensions of the framework are (1) time—continuity of care over time i.e. from the pre-pregnancy period through antenatal, delivery and postnatal periods for women, and care for children from the new-born period through adolescence; and (2) place—integrated service delivery provided by communities, first-level and referral health facilities [9,11]. Earlier studies on continuum of care mostly focussed on factors associated with uptake of CoC and its association with child health and child survival [5,34]. However, studies examining the spatial pattern of CoC are rare. In view of this the present study is the first attempt to examine the spatial pattern and determinants of full CoC at the district level. Districts are the smallest administrative units in India, and the level at which policies and programs are generally implemented. Thus, strength of the study lies in terms of district level coverage and spatial analysis methods to locate high-high (hotspots) and low-low (cold spots) of full CoC coverage in India. Moreover, the spatial lag and error model became apparent in explaining the predictors of full CoC coverage at the district level. In the SEM model modern contraceptive use and meeting with health worker have positive impact on the utilisation of CoC. Apart from this urbanization and education of women also have a positive impact on full CoC utilisation.

An interesting finding of the study is positive association between meeting health care workers (ASHA/ANM) in last 3 months and continuum of care completion. An important approach in the integrated services that address the entire continuum from the pregnancy to the postnatal period is training of the community health workers (CHWs) to encourage the mothers to access the various MCH services. Our study shows similar findings that involvement and contact with the community health workers has a positive association with CoC completion. Earlier findings in Indian context indicates that exposure to the ASHA is associated with an increased probability of women receiving at least one ANC and SBA [50]. The community health workers should ensure that all women receive high-quality, equitable and respectful care [51] which would facilitate the women to avail more services and prevent drop-out from availing full continuum of care. Due to several social, economic and cultural constraints women may limit the use of four or more ANC visits [52,53], the ASHA/ANM can play an important role in addressing several of these factors to encourage women, at the community level, to go for ANC visits. Mostly dropouts in the full CoC include dropout in SBA and PNC [54]. It is important to know who the providers of SBA and PNC are where these services are being delivered. Thus WHO endorses training the CHWs to conduct PNC checks at home where there are shortages of formal health workers [55].

The study also reveals the positive association between modern contraceptive use and continuum of care completion. It is widely recognized that family planning contributes towards reducing maternal mortality by restricting the number of births. Maternal health services do include an invaluable opportunity to educate mothers on family planning [56]. Earlier studies on integrating FP with MH services indicate increase in postpartum contraceptive use [57]. Family planning and maternal health services in most countries come as an integrated approach where women are educated about the post-partum contraceptive use in the 1^st^ ANC visit. However it was found that the proportion of women who received any information related to FP with ANC remained low in several countries across Latin America, the Caribbean and sub-Saharan Africa and India [57,58]. Contraceptive use and continuum of care completion should come as an integrated approach where use of contraception would lead to lesser fertility and hence more CoC completion in one hand and in other hand complete CoC would increase contraceptive use [59,60].

The study results indicate that level of urbanization is positively associated with full continuum of care. This finding corroborates with findings from earlier studies which indicate rural-urban differentials in utilization of maternal health care services [61,62]. Moreover earlier findings in Gambia, Indonesia and Haiti indicate that women living in the rural areas have a negative association with completion of continuum of care [63–65]. A number of factors determine the positive association between urbanization and uptake of full completion of care. These factors include better health infrastructure, nearness to hospitals, and availability of electricity, improved transportation, water and sanitation [16,66]. In contrast the rural mothers suffer from various disadvantages which includes transportation cost, low awareness about health-promoting behaviour, lack of health workers, poor infrastructure [67].

Our findings suggest that educational status of the mother and completion of continuum of care are positively associated. This association is extensive in the public health literature. Earlier studies have also indicated a positive association between education and uptake of maternal health care services in various countries [68,69]. With education, women are more aware and have greater choices and autonomy about their health care needs, which increases the likelihood of better utilization of maternal health care services [70–72].

Though earlier studies have indicated the various socio-economic and demographic determinants of continuum of care completion, but the studies ignored the spatial dimension. The spatial auto-correlation (*Lambda*) came out to be statistically significant in the spatial lag and error models, indicating that the relationship between continuum of care completion and independent variables at the district level may be misleading if spatial clustering is ignored. However, the study suffers from limitations as well. The first and foremost limitation is use of cross-sectional data which restricts to a particular reference time period and causality could not be assessed.

## Conclusion

Univariate and bivariate LISA provide good insight on the spatial clustering of the variables included in the analysis. Univariate LISA maps also help to identify the districts/geographical regions where more focus is needed for example the low-low clustering in complete CoC is seen in the states of Uttar Pradesh, Bihar and Madhya Pradesh and these areas need much focus with regards to MCH services. The study has also helped us to identify the outliers i.e. some regions of Central India have a higher percentage of Hindu women population and low completed CoC, while some parts of Jammu and Kashmir show a low-high association i.e. lower percentage of Hindu women and higher CoC uptake. However, it is difficult to conclude whether such relationships are attributable to specific behaviours, random variations or poor quality of data.

Future studies must focus on outliers to determine the reasons for the observed relationship.

The study suggests policymakers and stakeholders to take joint responsibility and increase the efforts for ensuring the completion of CoC by pregnant women. The spatial pattern indicates district/region specific targeted interventions and strategies may be needed for women of different socioeconomic status. The role of ASHA/ANM is very crucial and they should be trained more in order to encourage the mothers to avail full CoC. Thus the spatial analyses in this study incorporating different geographical regions exhibit an unexplored dimension in the context of CoC analysis in India.

## Supporting information

S1 Fig A to C: District Wise Distribution of A) Ante-Natal Care, B) Delivery through Skilled Birth Attendant and C) Post Natal Check-up within 48 hours of delivery among Mothers for Last Birth, India 2015–16.
(TIF)

S1 Table The number of observations and total fertility rate for 640 districts of India, NFHS 4 2015–16. (PDF)

S2 Table Detailed explanation on OLS and spatial models diagnostics. (PDF)
