# Supplementary figures and images for: Exploration of spatial clustering in maternal health continuum of care across districts of India: A geospatial analysis of demographic and health survey data
Source: PLoS One. 2022 Dec 15;17(12):e0279117. doi: 10.1371/journal.pone.0279117 (PMC9754170; doi:10.1371/journal.pone.0279117)

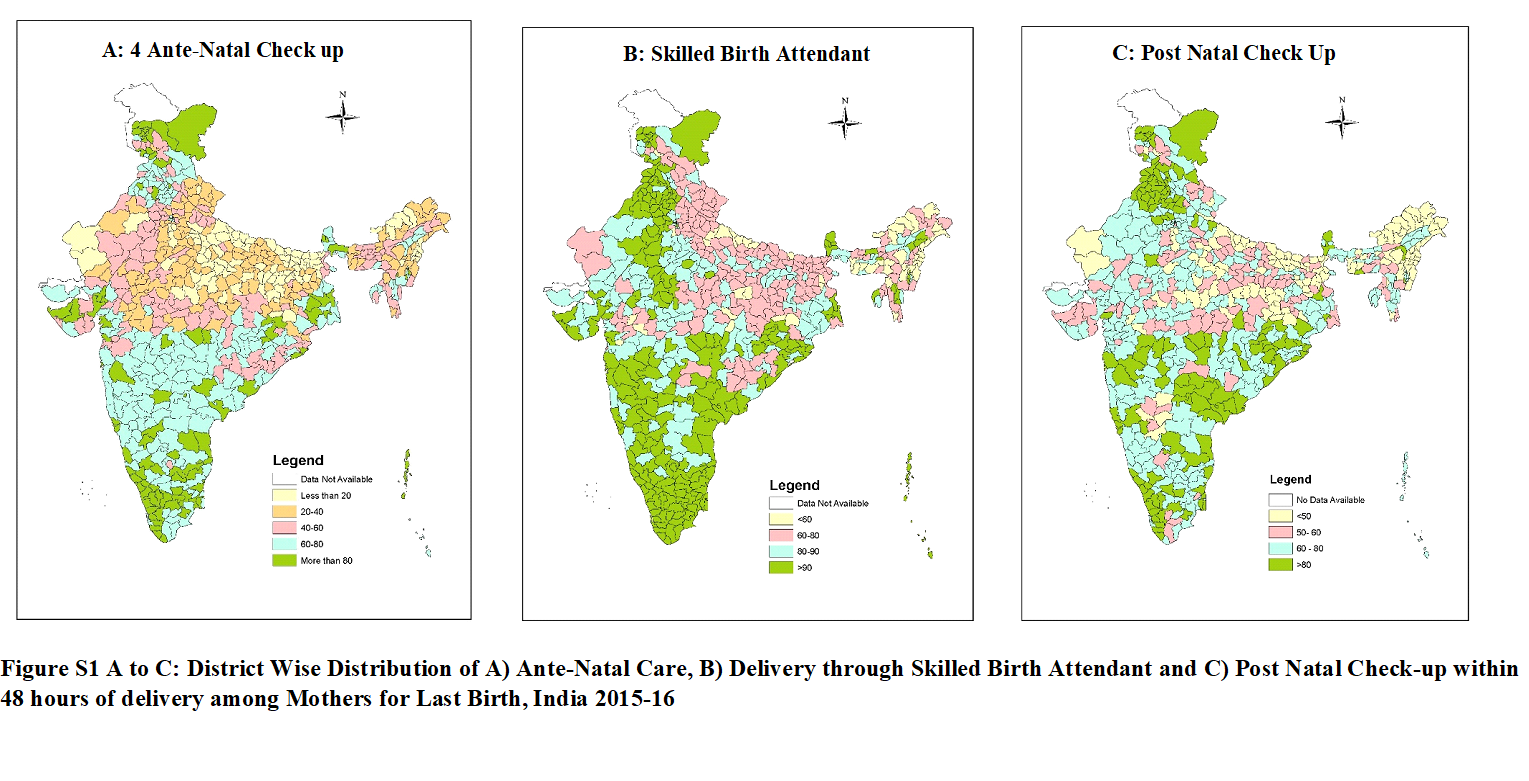

Supplement: S1 Fig — A to C: District Wise Distribution of A) Ante-Natal Care, B) Delivery through Skilled Birth Attendant and C) Post Natal Check-up within 48 hours of delivery among Mothers for Last Birth, India 2015–16. (TIF) [file pone.0279117.s001.tif]
