# Supplementary material for: Exploration of spatial clustering in maternal health continuum of care across districts of India: A geospatial analysis of demographic and health survey data
Source: PLoS One. 2022 Dec 15;17(12):e0279117. doi: 10.1371/journal.pone.0279117 (PMC9754170; doi:10.1371/journal.pone.0279117)
Supplement: S1 Table — (PDF) [file pone.0279117.s002.pdf]

Table S1: The Number of Observations and Total Fertility Rate for 640 districts of India, NFHS 4 2015-16

District	N	TFR	District	N	TFR	District	N	TFR
Kupwara	344	2.73	Bathinda	200	1.62	North	171	1.95
Badgam	278	1.79	Mansa	225	1.73	North east	206	2.00
Leh	242	2.17	Patiala	239	1.74	East	130	1.77
Kargil	308	2.35	Amritsar	243	1.62	New delhi	95	1.62
Punch	420	2.38	Tarn taran	232	1.71	Central	116	1.01
Rajouri	332	2.44	Rupnagar	197	1.71	West	124	1.30
Kathua	256	2.04	Sahibzada ajit singh	212	1.81	South west	167	2.29
Baramula	234	1.78	Shahid bhagat singh n	216	1.58	South	131	1.65
Bandipore	301	2.12	Barnala	185	1.54	Ganganagar	284	1.88
Srinagar	200	1.44	Chandigarh	150	1.57	Hanumangarh	245	1.84
Ganderbal	322	2.15	Uttarkashi	227	2.09	Bikaner	697	2.47
Pulwama	242	1.55	Chamoli	214	1.94	Churu	329	2.31
Shupiyan	263	1.70	Rudraprayag	191	2.00	Jhunjhunun	300	1.84
Anantnag	263	1.73	Tehri garhwal	262	1.95	Alwar	326	2.52
Kulgam	276	1.99	Dehradun	432	1.49	Bharatpur	375	3.54
Doda	271	2.89	Garhwal	207	1.98	Dhaulpur	382	3.12
Ramban	318	3.17	Pithoragarh	205	2.06	Karauli	331	2.99
Kishtwar	281	2.87	Bageshwar	264	2.03	Sawai madhopur	337	2.69
Udhampur	303	2.36	Almora	240	2.39	Dausa	297	2.32
Reasi	373	2.33	Champawat	259	2.07	Jaipur	609	2.03
Jammu	202	1.67	Nainital	519	1.96	Sikar	327	2.22
Samba	251	2.10	Udham singh nagar	599	2.17	Nagaur	300	2.14
Chamba	232	1.95	Hardwar	679	2.78	Jodhpur	620	2.35
Kangra	176	1.63	Panchkula	194	1.54	Jaisalmer	383	3.22
Lahul and spiti	135	1.79	Ambala	250	1.49	Barmer	368	3.61
Kullu	202	1.94	Yamunanagar	232	1.28	Jalor	374	3.08
Mandi	212	2.01	Kurukshetra	221	1.60	Sirohi	339	3.00
Hamirpur	189	1.82	Kaithal	281	2.10	Pali	285	2.21
Una	203	1.86	Karnal	275	2.17	Ajmer	634	2.14
Bilaspur	201	2.17	Panipat	210	1.56	Tonk	289	2.20
Solan	187	1.69	Sonipat	235	1.67	Bundi	261	2.52
Sirmaur	223	2.18	Jind	303	1.94	Bhilwara	259	2.22
Shimla	182	1.84	Fatehabad	288	1.91	Rajsamand	315	2.76
Kinnaur	150	1.91	Sirsa	340	2.18	Dungarpur	359	2.90
Gurdaspur	200	1.72	Hisar	245	2.08	Banswara	335	2.51
Kapurthala	182	1.31	Bhiwani	306	2.11	Chittaurgarh	233	1.91
Jalandhar	185	1.42	Rohtak	255	2.23	Kota	527	1.72
Hoshiarpur	179	1.68	Jhajjar	256	1.84	Baran	313	1.97
Sangrur	180	1.59	Mahendragarh	233	1.66	Jhalawar	264	1.87
Fatehgarh sahib	184	1.59	Rewari	253	1.87	Udaipur	324	2.84
Ludhiana	207	1.55	Gurgaon	257	1.93	Pratapgarh	329	2.59
Moga	222	1.85	Mewat	520	5.67	Saharanpur	709	2.72

Firozpur	208	1.72	Faridabad	241	2.15	Muzaffarnagar	364	3.10
Muktsar	204	1.47	Palwal	324	2.68	Bijnor	335	2.74
Faridkot	232	1.36	North west	118	1.59	Moradabad	693	2.95
**District**	**N**	**TFR**	**District**	**N**	**TFR**	**District**	**N**	**TFR**
Rampur	354	2.94	Shrawasti	499	4.40	Munger	354	3.13
Jyotiba phule nagar	362	2.95	Balrampur	460	3.38	Lakhisarai	460	3.36
Meerut	932	2.27	Gonda	437	3.31	Sheikhpura	473	3.62
Baghpat	302	2.24	Siddharth nagar	461	3.41	Nalanda	372	3.21
Ghaziabad	645	2.42	Basti	400	3.01	Patna	757	2.68
Gautam buddha nagar	763	2.61	Sant kabir nagar	410	3.05	Bhojpur	414	2.82
Bulandshahr	373	2.92	Mahrajganj	407	2.82	Buxar	429	2.92
Aligarh	729	2.85	Gorakhpur	331	2.38	Kaimur (bhabua)	397	3.43
Mahamaya nagar	352	2.74	Kushinagar	421	3.00	Rohtas	434	2.88
Mathura	366	2.88	Deoria	348	2.43	Aurangabad	368	2.66
Agra	739	2.80	Azamgarh	347	2.45	Gaya	534	3.35
Firozabad	700	2.78	Mau	368	2.66	Nawada	425	3.06
Mainpuri	325	2.69	Ballia	360	2.84	Jamui	504	3.65
Budaun	404	3.73	Jaunpur	378	2.72	Jehanabad	372	2.96
Bareilly	645	2.52	Ghazipur	345	2.80	Arwal	432	3.26
Pilibhit	355	2.73	Chandauli	414	2.75	North district	184	1.31
Shahjahanpur	365	3.48	Varanasi	626	2.22	West district	172	1.05
Kheri	381	3.38	Sant ravidas nagar (b	427	3.00	South district	157	1.09
Sitapur	349	3.32	Mirzapur	361	2.91	East district	386	1.26
Hardoi	321	3.03	Sonbhadra	329	2.83	Tawang	199	2.55
Unnao	314	2.74	Etah	368	3.02	West kameng	222	2.55
Lucknow	388	1.58	Kanshiram nagar	391	3.50	East kameng	295	3.51
Rae bareli	281	2.48	Pashchim champaran	389	3.91	Papumpare	481	1.91
Farrukhabad	367	3.24	Purba champaran	469	4.16	Upper subansiri	257	2.35
Kannauj	366	3.06	Sheohar	451	4.27	West siang	168	1.76
Etawah	339	2.51	Sitamarhi	442	3.73	East siang	170	1.22
Auraiya	288	2.60	Madhubani	424	3.44	Upper siang	149	1.14
Kanpur dehat	272	2.54	Supaul	501	3.99	Changlang	248	1.92
Kanpur nagar	418	1.64	Araria	451	3.93	Tirap	259	1.63
Jalaun	206	2.00	Kishanganj	453	3.78	Lower subansiri	159	1.79
Jhansi	441	2.05	Purnia	468	3.91	Kurung kumey	306	3.16
Lalitpur	240	2.31	Katihar	402	3.65	Dibang valley	182	1.84
Hamirpur	236	2.34	Madhepura	493	3.93	Lower dibang valley	235	2.01
Mahoba	255	2.43	Saharsa	467	4.24	Lohit	303	2.45
Banda	212	2.67	Darbhanga	431	3.73	Anjaw	225	1.72
Chitrakoot	361	3.36	Muzaffarpur	370	3.15	Mon	380	3.83
Fatehpur	244	2.32	Gopalganj	396	2.55	Mokokchung	118	1.92
Pratapgarh	300	2.30	Siwan	424	2.77	Zunheboto	176	2.70
Kaushambi	333	3.27	Saran	396	3.26	Wokha	170	2.54
Allahabad	349	2.46	Vaishali	465	3.21	Dimapur	549	2.14
Bara banki	300	2.60	Samastipur	410	3.72	Phek	258	3.19
Faizabad	343	2.63	Begusarai	448	3.28	Tuensang	284	3.92

Ambedkar nagar	386	2.36	Khagaria	471	3.89	Longleng	242	2.61
Sultanpur	306	2.74	Bhagalpur	449	3.45	Kiphire	277	3.41
Bahraich	445	4.22	Banka	427	3.36	Kohima	380	1.92
**District**	**N**	**TFR**	**District**	**N**	**TFR**	**District**	**N**	**TFR**
Peren	302	3.26	Cachar	291	2.29	Bilaspur	393	2.71
Senapati (excluding 3	365	3.36	Karimganj	365	2.78	Kabirdham	315	2.36
Tamenglong	393	3.81	Hailakandi	329	2.64	Rajnandgaon	402	2.67
Churachandpur	302	3.07	Bongaigaon	338	2.21	Durg	550	1.85
Bishnupur	722	2.41	Chirang	333	2.53	Raipur	577	2.07
Thoubal	731	2.60	Kamrup	300	1.74	Mahasamund	275	2.15
Imphal west	627	2.28	Kamrup metropolitan	206	1.59	Dhamtari	259	1.70
Imphal east	657	2.18	Nalbari	285	1.89	Uttar bastar kanker	264	1.80
Ukhrul	301	3.43	Baksa	339	2.02	Bastar	329	2.40
Chandel	331	3.04	Darrang	363	2.38	Narayanpur	455	2.57
Mamit	312	2.67	Udalguri	316	2.12	Dakshin bastar dantew	296	2.20
Kolasib	563	2.70	Darjiling	178	1.53	Bijapur	421	2.58
Aizawl	268	2.05	Jalpaiguri	212	1.69	Sheopur	332	2.85
Champhai	650	2.97	Koch bihar	215	1.85	Morena	309	2.64
Serchhip	548	2.27	Uttar dinajpur	333	2.65	Bhind	309	2.62
Lunglei	443	1.97	Dakshin dinajpur	196	1.67	Gwalior	616	2.20
Lawngtlai	290	2.27	Maldah	298	2.44	Datia	289	2.44
Saiha	607	2.77	Murshidabad	292	1.96	Shivpuri	320	2.47
West tripura	441	1.57	Birbhum	228	1.74	Tikamgarh	285	2.07
South tripura	196	1.38	Barddhaman	223	1.55	Chhatarpur	313	2.76
Dhalai	249	1.70	Nadia	222	1.81	Panna	270	2.51
North tripura	283	2.35	North twenty four par	209	1.46	Sagar	305	2.76
West garo hills	313	1.88	Hugli	192	1.52	Damoh	278	2.15
East garo hills	426	2.93	Bankura	227	1.72	Satna	252	2.29
South garo hills	312	1.69	Puruliya	298	2.26	Rewa	308	2.44
West khasi hills	450	4.43	Haora	219	1.60	Umaria	290	2.51
Ribhoi	438	3.69	Kolkata	148	1.41	Neemuch	293	1.97
East khasi hills	665	2.98	South twenty four par	294	2.09	Mandsaur	341	2.11
Jaintia hills	515	4.48	Paschim medinipur	225	1.60	Ratlam	302	2.27
Kokrajhar	299	2.18	Purba medinipur	250	1.72	Ujjain	702	2.13
Dhubri	375	2.80	Garhwa	357	3.40	Shajapur	321	2.25
Goalpara	385	2.81	Chatra	425	3.30	Dewas	329	2.45
Barpeta	370	2.36	Kodarma	347	3.04	Dhar	348	2.23
Morigaon	369	2.97	Giridih	396	3.01	Indore	615	1.90
Nagaon	329	2.83	Deoghar	392	2.94	Khargone (west nimar)	326	2.23
Sonitpur	260	1.70	Godda	314	3.13	Barwani	451	3.08
Lakhimpur	296	2.16	Sahibganj	370	3.55	Rajgarh	310	2.68
Dhemaji	365	2.29	Pakur	374	3.12	Vidisha	307	2.80
Tinsukia	281	1.96	Dhanbad	532	1.77	Bhopal	276	1.85
Dibrugarh	274	1.71	Bokaro	527	2.11	Sehore	298	2.47
Sivasagar	272	1.89	Lohardaga	298	2.62	Raisen	338	2.45
Jorhat	262	1.57	Purbi singhbhum	448	1.65	Betul	274	1.81

Golaghat	281	1.73	Palamu	320	3.04	Harda	329	2.20
Karbi anglong	329	2.25	Latehar	314	2.80	Hoshangabad	578	2.08
Dima hasao	322	2.59	Hazaribagh	347	2.37	Katni	256	2.14
**District**	**N**	**TFR**	**District**	**N**	**TFR**	**District**	**N**	**TFR**
Jabalpur	502	1.85	Dadra & nagar haveli	244	2.32	Visakhapatnam	143	1.55
Narsimhapur	245	1.82	Nandurbar	243	2.15	East godavari	164	1.58
Dindori	273	2.22	Dhule	222	2.17	West godavari	128	1.64
Mandla	249	1.95	Jalgaon	192	2.26	Krishna	145	1.45
Chhindwara	273	1.93	Buldana	221	1.80	Guntur	145	1.81
Seoni	257	1.79	Akola	247	1.84	Prakasam	196	2.12
Balaghat	275	2.15	Washim	231	1.98	Sri potti sriramulu n	182	1.94
Guna	373	2.50	Amravati	186	1.64	Y.s.r.	201	2.12
Ashoknagar	326	2.56	Wardha	160	1.55	Kurnool	254	2.21
Shahdol	245	2.09	Nagpur	225	1.58	Anantapur	173	1.91
Anuppur	280	2.27	Bhandara	159	1.57	Chittoor	170	1.99
Sidhi	354	3.02	Gondiya	208	1.94	Belgaum	222	2.06
Singrauli	409	3.09	Gadchiroli	171	1.50	Bagalkot	233	2.02
Jhabua	365	3.52	Chandrapur	172	1.84	Bijapur	230	1.92
Alirajpur	499	3.52	Yavatmal	204	1.97	Bidar	217	1.97
Khandwa (east nimar)	344	2.47	Nanded	302	2.19	Raichur	266	2.08
Burhanpur	667	2.60	Hingoli	241	2.17	Koppal	233	2.01
Kachchh	235	2.31	Parbhani	261	2.09	Gadag	196	1.42
Banaskantha	278	2.72	Jalna	273	2.45	Dharwad	201	1.98
Patan	246	1.95	Aurangabad	243	2.15	Uttara kannada	174	1.68
Mahesana	211	2.20	Nashik	251	2.04	Haveri	192	1.61
Sabarkantha	242	2.18	Thane	179	1.80	Bellary	257	2.29
Gandhinagar	227	2.17	Mumbai suburban	138	1.51	Chitradurga	160	1.38
Ahmadabad	176	1.94	Mumbai	135	1.76	Davanagere	195	2.13
Surendranagar	231	1.96	Raigarh	178	1.96	Shimoga	168	1.71
Rajkot	194	1.69	Pune	168	1.67	Udupi	178	1.55
Jamnagar	215	1.73	Ahmadnagar	208	2.10	Chikmagalur	147	1.67
Porbandar	244	1.71	Bid	230	2.31	Tumkur	162	1.46
Junagadh	215	1.84	Latur	267	2.39	Bangalore	185	1.53
Amreli	159	1.68	Osmanabad	192	2.11	Mandya	144	1.71
Bhavnagar	209	1.80	Solapur	221	1.84	Hassan	160	1.80
Anand	217	2.09	Satara	176	2.15	Dakshina kannada	176	1.86
Kheda	237	2.36	Ratnagiri	152	1.93	Kodagu	183	1.98
Panchmahal	251	2.30	Sindhudurg	132	1.51	Mysore	180	1.86
Dohad	248	2.80	Kolhapur	173	1.46	Chamarajanagar	175	1.38
Vadodara	216	2.09	Sangli	182	1.76	Gulbarga	254	2.12
Narmada	263	2.29	Adilabad	179	1.84	Yadgir	265	2.30
Bharuch	213	2.23	Nizamabad	184	2.05	Kolar	203	1.83
The dangs	278	2.93	Karimnagar	156	1.43	Chikkaballapura	162	1.70
Navsari	181	1.78	Medak	182	1.74	Bangalore rural	205	1.69
Valsad	210	1.90	Hyderabad	193	1.83	Ramanagara	153	1.51
Surat	234	1.77	Rangareddy	182	1.65	North goa	180	1.62

Tapi	197	1.62	Mahbubnagar	217	2.53	South goa	167	1.73
Diu	180	2.01	Nalgonda	181	1.82	Lakshadweep	262	1.82
Daman	151	1.60	Vizianagaram	192	1.82	Kasaragod	182	1.61
**District**	**N**	**TFR**	**District**	**N**	**TFR**			
Kannur	154	1.67	Yanam	212	1.61			
Wayanad	195	1.61	Puducherry	209	1.64			
Kozhikode	155	1.46	Mahe	240	1.62			
Malappuram	228	1.70	Karaikal	218	1.95			
Palakkad	179	1.61	Nicobars	189	1.70			
Thrissur	126	1.44	North & middle andama	169	1.46			
Ernakulam	132	1.58	South andaman	168	1.41			
Idukki	111	1.25						
Kottayam	141	1.51						
Alappuzha	125	1.42						
Pathanamthitta	120	1.60						
Kollam	141	1.46						
Thiruvananthapuram	139	1.65						
Thiruvallur	163	1.45						
Chennai	197	0.99						
Kancheepuram	180	1.40						
Vellore	214	1.81						
Tiruvannamalai	176	1.73						
Viluppuram	221	1.82						
Salem	220	1.83						
Namakkal	199	1.56						
Erode	209	1.67						
The nilgiris	198	1.57						
Dindigul	206	1.74						
Karur	238	1.98						
Tiruchirappalli	184	1.86						
Perambalur	178	1.60						
Ariyalur	158	1.71						
Cuddalore	217	1.88						
Nagapattinam	198	1.58						
Thiruvarur	192	1.88						
Thanjavur	195	1.92						
Pudukkottai	217	1.96						
Sivaganga	210	1.94						
Madurai	170	1.82						
Theni	151	1.53						
Virudhunagar	150	1.98						
Ramanathapuram	193	2.09						
Thoothukkudi	166	1.74						
Tirunelveli	146	1.67						
Kanniyakumari	206	1.97						
Dharmapuri	196	1.74						

Krishnagiri	231	1.75			
Coimbatore	212	1.78			
Tiruppur	190	1.65			
