# Supplementary material for: Exploration of spatial clustering in maternal health continuum of care across districts of India: A geospatial analysis of demographic and health survey data
Source: PLoS One. 2022 Dec 15;17(12):e0279117. doi: 10.1371/journal.pone.0279117 (PMC9754170; doi:10.1371/journal.pone.0279117)
Supplement: S2 Table — (PDF) [file pone.0279117.s003.pdf]

Table S2: Diagnostic Test for Testing Spatial Dependency

**Test for Spatial dependence**			
TEST	MI/DF	VALUE	PROB
Moran's I (error)	0.4989	17.2857	0
Lagrange Multiplier (lag)	1	144.1606	0
Robust LM (lag)	1	0.9883	0.32016
Lagrange Multiplier (error)	1	280.4486	0
Robust LM (error)	1	137.2763	0
Lagrange Multiplier (SARMA)	2	281.4369	0
